# Facial attractiveness and preference of sexual dimorphism: A comparison across five populations

**DOI:** 10.1017/ehs.2021.33

**Published:** 2021-07-02

**Authors:** Vojtěch Fiala, Vít Třebický, Farid Pazhoohi, Juan David Leongómez, Petr Tureček, S. Adil Saribay, Robert Mbe Akoko, Karel Kleisner

**Affiliations:** 1 Department of Philosophy and History of Science, Faculty of Science, Charles University, Viničná 7, 128 44 Prague, Czech Republic; 2 Faculty of Physical Education and Sport, Charles University, Prague, Czech Republic; 3 Department of Psychology, University of British Columbia, 2136 West Mall, Vancouver, British Columbia, V6T 1Z4, Canada; 4 Human Behaviour Laboratory, Faculty of Psychology, Universidad El Bosque, Bogota, Colombia; 5 Department of Psychology, Kadir Has University, Istanbul, Turkey; 6 Department of Communication and Development Studies, University of Bamenda, Cameroon

**Keywords:** Human face, skin luminance, sexual dimorphism, averageness, geometric morphometrics

## Abstract

Despite intensive research, evolutionary psychology has not yet reached a consensus regarding the association between sexual dimorphism and attractiveness. This study examines associations between perceived and morphological facial sexual dimorphism and perceived attractiveness in samples from five distant countries (Cameroon, Colombia, Czechia, Iran and Turkey). We also examined possible moderating effects of skin lightness, averageness, age, body mass and facial width. Our results suggest that in all samples, women's perceived femininity was positively related to their perceived attractiveness. Women found perceived masculinity in men attractive only in Czechia and Colombia, two distant populations. The association between perceived sexual dimorphism and attractiveness is thus potentially universal only for women. Across populations, morphological sexual dimorphism and averageness are not universally associated with either perceived facial sexual dimorphism or attractiveness. With our exploratory approach, results highlight the need for control of which measure of sexual dimorphism is used (perceived or measured) because they affect perceived attractiveness differently. Morphological averageness and sexual dimorphism are not good predictors of perceived attractiveness. It is noted that future studies should use samples from multiple populations to allow for identification of specific effects of local environmental and socioeconomic conditions on preferred traits in unmanipulated local facial stimuli.

## Graphical abstract

**Figure:**
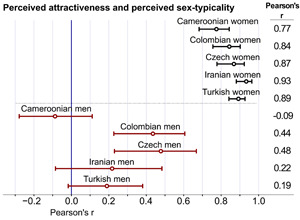

**Social media summary:** Morphological sexual dimorphism is not universally associated with perceived facial sexual dimorphism and attractiveness

## 1. Introduction

According to the signalling theory, facial traits which are perceived as attractive are considered honest cues of biological fitness (Gangestad & Scheyd, 2005; Thornhill & Gangestad, 1999; Kościński, 2007, 2008), in particular healthiness and viability (Henderson et al., 2016; Rhodes et al., 2003), hormone-based development of secondary sexual characteristics and fertility (Law Smith et al., 2006; Rantala et al., 2012; Whitehouse et al., 2015). They also provide specific cues to psychological characteristics that are important in partnership and childbearing, such as faithfulness (Boothroyd et al., 2008) and willingness to cooperate (Stirrat & Perrett, 2010).

Although there is some variation in the perception of facial attractiveness between individuals belonging to the same local population (Bronstad & Russell, 2007; Germine et al., 2015; Hönekopp, 2006; Kramer et al., 2018), people from similar cultural backgrounds tend to perceive facial attractiveness similarly (Kowner & Ogawa, 1995; Langlois et al., 2000; Little & Hancock, 2002; Strzałko & Kaszycka, 1992).

While there is some evidence that supports a hypothesis of cross-cultural consensus on attractiveness ratings (Burke et al., 2013; Coetzee et al., 2014; Langlois et al., 2000), a growing number of studies report mixed results regarding agreement between samples from distant countries (Apicella et al., 2007; Jones & Hill, 1993; Sorokowski, Kościński, et al., 2013; Zebrowitz et al., 2012). It is not, however, the case that members of distant populations either agree or disagree entirely on which traits are perceived as attractive. Instead, some facial traits are preferred across distant samples while others are not. For example, youthfulness (Buss, 1989; Maestripieri et al., 2014; McLellan & McKelvie, 1993) and average facial traits (Deffenbacher et al., 1998; Komori et al., 2009; Rhodes et al., 2001; cf. Apicella et al., 2007) are preferred universally.

Aside from that, there is a cross-cultural agreement concerning preferences regarding facial skin colouration. Lighter-skinned women are perceived as more attractive than darker-skinned women within a given population (Aoki, 2002; Badaruddoza, 2007; Carrito et al., 2016; Dixson et al., 2010; van den Berghe & Frost, 1986; Wagatsuma, 1967), but for some dark-skinned populations, the results are less conclusive (Dixson et al., 2017; Sorokowski, Sorokowska, et al., 2013). Moreover, in a cross-cultural comparison, skin colouration variance is an important trait for ratings of ethnic typicality and attractiveness for African raters, while it is less important for ratings made by Europeans regardless of the ethnic origin of the presented stimulus faces (Coetzee et al., 2014; Strom et al., 2012; Kleisner et al., 2017).

On the other hand, preference for facial sexual dimorphism (how well the development of facial shape and colouration represents features typical for a given sex) varies substantially across populations from distant countries, especially with respect to male faces (DeBruine, Jones, Crawford, et al., 2010; Marcinkowska et al., 2019, and citations below).

Such differences in attractiveness perception between populations from distant countries contradict the assumption that facial attractiveness serves as a cue of biological quality. A plausible evolutionary-based explanation is that in populations that live in different environments, preferred traits may vary because different characteristics are optimal for survival and reproduction under different environmental conditions (DeBruine, Jones, Crawford, et al., 2010; Lee & Zietsch, 2011; Little et al., 2007).

### 1.1. Morphological and perceived sexual dimorphism and attractiveness

During ontogeny, the facial traits of men and women gradually diverge owing to the action of sex steroids (Marečková et al., 2011; Whitehouse et al., 2015). As a result, adult faces acquire sexually dimorphic features (Hausman, 1999; Mooradian et al., 1987; Worthman, 1995).

Higher levels of perceived feminine characteristics in women's faces are associated with higher perceived attractiveness, as evidenced by previous studies that used non-manipulated women's faces (Foo, Simmons, et al., 2017; Muñoz-Reyes et al., 2015; Scott et al., 2010), manipulated composite female facial stimuli (Perrett et al., 1994, 1998; Rhodes et al., 2000; Smith et al., 2009) and even manipulated individual women's faces (Mogilski & Welling, 2017; for a review, see Rhodes, 2006). Women with more sex-typical (more feminine) facial features were also shown to have relatively higher oestrogen levels (Durante & Li, 2009; Law Smith et al., 2006; Probst et al., 2016). There is evidence from a US sample to the effect that fertility is positively associated with oestrogen levels (Lipson & Ellison, 1996). On the other hand, in deprived, poorer and rural populations – where women generally have lower sex hormone levels – fertility is relatively high (Vitzhum, 2009; Vitzhum et al., 2002). It has also been shown that the preference for femininity in women's faces is weaker in deprived populations and populations with worse health indices (De Barra et al., 2013; Marcinkowska et al., 2014; Penton-Voak et al., 2004)**.**

With respect to preference for men's facial sexual dimorphism, the evidence is mixed. Some studies report preferences for less masculine features in men's faces (Perrett et al., 1998; Rhodes et al., 2000), others show preferences for more masculine features (Foo, Simmons, et al., 2017; Johnston et al., 2001; Peters et al., 2008; Skrinda et al., 2014). A number of studies found preferences for neither masculine nor feminine features in male faces (Mogilski & Welling, 2017; Penton-Voak & Chen, 2004; Scott et al., 2010; Stephen et al., 2012). It has been suggested that methodological differences in stimuli manipulation are at least in part responsible for such mixed results (Rennels et al., 2008; Rhodes, 2006), but it is also possible that distant populations actually differ in preferred male facial traits. The usual approach to addressing this variation in results is to test adaptive hypotheses on fitness outcomes of preference of certain traits across various populations (Brooks et al., 2011; DeBruine et al., 2011; DeBruine, Jones, Crawford, et al., 2010).

Masculine features may be cues to health. Men with masculine facial features have been considered more immunocompetent (Foo, Nakagawa, et al., 2017; cf. Nowak et al., 2018; Rantala et al., 2012) and healthier than their less masculine peers (Rhodes et al., 2003; Thornhill & Gangestad, 2006; see also Moore et al., 2011; Rantala et al., 2012). It is thus hypothesised that by preferring more masculine men, women try to increase their chances of acquiring a healthier mate who would have more immunocompetent offspring.

Moreover, formidability and resource holding potential, i.e. an individual's willingness to engage in conflict over resources stemming from his/her ability to obtain or withdraw resources from a rival (Třebický et al., 2019), are also cued by masculine facial features (Boothroyd et al., 2007; Carre & McCormick, 2008; Stirrat & Perrett, 2010; Swaddle & Reierson, 2002; Třebický et al., 2015). Women who are endangered by unequal resource distribution (e.g. owing to economic inequality) may prefer more masculine men because such men are more likely to obtain resources for their families (Brooks et al., 2011; Little et al., 2013). Moreover, the preference for masculine men seems adaptive because a formidable and dominant partner can better protect his mate against violence and harm (Ryder et al., 2016).

Nevertheless, masculinity is also linked to the risk of testosterone-associated antisocial behaviours (van Bokhoven et al., 2006), higher divorce probability (Booth et al., 1993; Mazur & Michalek, 1998) and low partner fidelity (Penton-Voak & Chen, 2004; Polo et al., 2019), which may all have a negative impact on parental investment. All in all, women seem to face a trade-off between choosing a masculine, immunocompetent and formidable mate, who could also harm and/or leave her, or a less masculine but cooperative and more nurturing mate. Such a trade-off may lack a universal solution across populations from distant countries.

### 1.2. The current study

Research on differences in the association between sexual dimorphism and perceived attractiveness usually builds on a single set of manipulated facial stimuli, which are then used for a number of sets of raters from distant populations in a hypothesis-driven research paradigm (DeBruine, Jones, Crawford, et al., 2010; Marcinkowska et al., 2019; Scott et al., 2014). It could be objected, however, that manipulation of sexual dimorphism may affect stimuli features in a way that need not be ecologically relevant to all of the investigated populations. Moreover, evaluation of faces by raters from a visually distinctive population may cause people to perform worse on trait attribution (Anzures et al., 2013). The use of local faces therefore has clear advantages.

There are multiple evolutionary-based hypotheses which aim to explain both the consensus and differences in preferences of various facial traits across people from distant populations (see above). Moreover, it is not clear whether raters across distant populations prefer similar traits in local faces and whether they interpret sexually dimorphic facial traits similarly.

We have therefore conducted a set of non-confirmatory analyses (following Scheel et al., 2020; see also Nakamura & Watanabe, 2019) on the association between perceived facial attractiveness, perceived and measured sexual dimorphism and other facial traits across populations from five distant countries (Cameroon, Colombia, Czechia, Iran and Turkey). In each country, we used only facial stimuli collected within the local population. Using various facial feature metrics and path analysis, we also explored sample-specific patterns of serial and parallel mediation among the predictors of perceived characteristics. Specifically, we used predictors that may affect perceived attractiveness, perceived sexual dimorphism and measured sexual dimorphism, and mediate their relationship, namely morphological averageness, skin lightness, age, body mass index (BMI) and facial width to height ratio (fWHR) (see Supplementary Material, Sections S1.1–S1.4).

To avoid any confounding effects of stimuli manipulation on trait attribution (DeBruine, Jones, Smith, et al., 2010; see also Kleisner et al., 2019; Rennels et al., 2008), we used unmanipulated facial stimuli. Moreover, we used two measures of facial sexual dimorphism: (a) perceived sex-typicality (perceived femininity in women and perceived masculinity in men); and (b) sexual shape dimorphism of facial shape calculated from landmark-based geometric morphometrics. According to a recent study, these two measures of facial sexual dimorphism are only moderately correlated (Mitteroecker et al., 2015), presumably because perceived sex-typicality is also affected by skin lightness across populations (Carrito & Semin, 2019; van den Berghe & Frost, 1986).

Although this study is of an exploratory nature, we made several predictions (see Table 3a): based on previous studies on the association between perceived and morphological sexual dimorphism (Komori et al., 2011; Mitteroecker et al., 2015), we expect that perceived and measured sexual dimorphism will be correlated in every sampled population positively, albeit weakly (*r* ≈ 0.3). Moreover, morphological averageness of facial configurations should be moderately positively associated with perceived attractiveness (Jones & Jaeger, 2019).

Skin lightness may affect perceived sex-typicality and attractiveness mainly in Cameroon, given that people of sub-Saharan African origin are more sensitive to facial skin colouration and lightness variability as a cue for both facial attractiveness (Kleisner et al., 2017) and ethnic typicality (Strom et al., 2012). Moreover, skin lightness has also been associated with perceived male sex-typicality and healthiness in previous research based on people of European origin (Carrito & Semin, 2019). Skin lightness should therefore negatively relate to perceived masculinity in male faces in all five samples.

We further predict that perceived femininity of women's faces will be associated with higher ratings of attractiveness across all five population samples (Cameroon, Colombia, Czechia, Iran and Turkey) regardless of their mutual distance and eventual cultural differences. It has been demonstrated that perceived sex-typicality of women is an important component of their perceived facial attractiveness. We therefore predict a strong association between perceived attractiveness and perceived femininity in women in samples from all of the five populations (*r* ≈ 0.8; see Foo, Simmons, et al., 2017; Koehler et al., 2004).

Concerning the preference for male sex-typicality, there is substantial disagreement across populations from distant countries (Marcinkowska et al., 2019) and even between same-country samples recruited from or primed to different socioeconomic conditions (DeBruine et al., 2011; Little, Cohen, et al., 2007). This suggests the existence of various factors that may shape masculinity preferences uniquely in each country. We sampled populations from only five countries, which is why we have only five data points on the cross-population level and therefore cannot anticipate which of those forces would drive preferences in our samples. All predictions are summarised in Table 3a.

## 2. Methods

For detailed descriptions of stimuli acquisition, measurements, rating procedures and analyses, see Supplementary Materials in the OSF entry for this study at https://osf.io/va8pg/?view_only=021e11321855463f82fc64e6ceb5716a.

This study was approved by the Institutional Review Board of Charles University, Faculty of Science. The photographed individuals and raters were informed about the purpose of data collection. Photographed individuals signed informed consent and raters consented by clicking ‘I agree’ in the questionnaire.

### 2.1. Facial photos acquisition

As stimuli, we collected a total of 709 standardised frontal facial photographs (357 men and 352 women) from five countries (Cameroon, Colombia, Czechia, Iran and Turkey). Data about age, height and body weight were collected from participants in Cameroon, Colombia, Czechia and Turkey, while only information about age was collected from Iranian participants. We used a pre-existing available photograph dataset of Iranian faces that has been acquired prior to the decision to use those photographs in this research and which lacked the information on the height and weight of the models. The effect of relative weight therefore could not be tested in the Iranian sample. The number of stimuli per sample and sex and samples’ descriptives are summarised in Table 1.

**Table 1.** Descriptive statistics of the stimuli sample

Sample	*N*	Age (mean ± SD)	BMI (mean ± SD)	CIELab L* (mean ± SD)	fWHR (mean ± SD)	SShD (mean ± SD)
Cameroonian women	100	22.01 ± 3.87	24.79 ± 4.56	38.88 ± 5.55	2.13 ± 0.16	−0.0119 ± 0.0147
Cameroonian men	100	22.00 ± 2.61	23.11 ± 2.09	34.65 ± 5.23	2.10 ± 0.16	0.0119 ± 0.0130
Colombian women	66	20.71 ± 2.77	22.65 ± 3.15	67.37 ± 4.05	2.00 ± 0.12	−0.0206 ± 0.0221
Colombian men	72	20.38 ± 2.42	22.92 ± 2.80	58.72 ± 5.31	1.99 ± 0.13	0.0189 ± 0.0258
Czech women	50	23.64 ± 4.33	22.16 ± 2.90	60.26 ± 4.27	1.95 ± 0.13	−0.0189 ± 0.0185
Czech men	50	24.04 ± 3.92	22.38 ± 2.27	58.16 ± 2.32	1.89 ± 0.13	0.0189 ± 0.0156
Iranian women	43	20.63 ± 1.09	NA	65.62 ± 3.74	1.78 ± 0.11	−0.0199 ± 0.0210
Iranian men	44	20.64 ± 1.60	NA	57.33 ± 5.76	1.81 ± 0.14	0.0195 ± 0.0173
Turkish women	93	21.20 ± 1.51	20.75 ± 2.92	80.55 ± 2.74	1.97 ± 0.11	−0.0223 ± 0.0148
Turkish men	91	21.52 ± 1.95	23.64 ± 3.51	77.90 ± 3.36	2.01 ± 0.13	0.0231 ± 0.0153
Sample	*N*	Avrg (mean ± SD)	Fem/masc (mean ± SD)	Fem/masc (ICC)	Attr (mean ± SD)	Attr (ICC)
Cameroonian women	100	0.0582 ± 0.0131	4.67 ± 0.70	0.94/0.97^a^	3.08 ± 0.65	0.91/0.96^a^
Cameroonian men	100	0.0571 ± 0.0121	5.52 ± 0.59	0.95/0.96^a^	3.38 ± 0.76	0.92/0.96^a^
Colombian women	66	0.0523 ± 0.0121	4.77 ± 1.09	0.99 (ICC 1,*k*)	3.26 ± 0.90	0.99 (ICC 1,*k*)
Colombian men	72	0.0561 ± 0.0168	4.84 ± 0.75	0.98 (ICC 1,*k*)	2.22 ± 0.54	0.98 (ICC 1,*k*)
Czech women	50	0.0542 ± 0.0128	3.86 ± 0.86	0.97	2.68 ± 0.86	0.97
Czech men	50	0.0544 ± 0.0118	4.07 ± 0.80	0.99	2.78 ± 0.87	0.99
Iranian women	43	0.0556 ± 0.0134	3.52 ± 0.55	0.92	2.03 ± 0.44	0.93
Iranian men	44	0.0585 ± 0.0152	4.34 ± 0.58	0.90	1.92 ± 0.46	0.92
Turkish women	93	0.0488 ± 0.0105	3.93 ± 0.81	0.95 (ICC 1,*k*)	2.89 ± 0.94	0.96 (ICC 1,*k*)
Turkish men	91	0.0526 ± 0.0113	4.44 ± 0.67	0.94 (ICC 1,*k*)	2.41 ± 0.76	0.95 (ICC 1,*k*)

fWHR, Facial width to height ratio; BMI, body mass index; CIELab *L*, skin lightness; SShD, sexual shape dimorphism (measured facial sexual dimorphism); Avrg, morphometrical averageness; Fem/masc, perceived femininity(women)/masculinity(men); Attr, perceived attractiveness; SD, standard deviation; ICC, intraclass correlation coefficient (measure of inter-rater agreement), ICC (3,*k*) if not otherwise stated. ^a^ Cameroonian samples from 2013 and 2016 were rated separately, ICCs are as follows: 2013 Sample/2016 Sample

The Cameroonian stimuli were collected in two separate runs (2013 and 2016) and these two sets were also rated separately. For the purpose of this study, the two Cameroonian samples were combined, but analyses of the separate samples yielded results similar to analyses of the combined sample (the alternative analyses are presented in Table S3 and Figure S5 in the Supplementary Material.).

The photographs were taken with digital cameras, using external light sources and homogeneous white or grey backgrounds. Lighting conditions were not standardised across the samples but were uniform within each sample (each country). All participants were asked to remove their glasses, facial jewellery, other adornments or cosmetics, adopt a neutral facial expression and look directly into the camera. All women in the Iranian set wore a hijab that covered hair, ears and part of their cheeks.

All photographs were adjusted to set the eyes horizontally at the same height and leave approximately the same length of neck visible. They were subsequently cropped and exported at ~500 × 700 px resolution; see Section S2.1 in the Supplementary Material for further details of photo acquisition.

The stimuli were sampled by convenience and they do not represent the populations as a whole. Nonetheless, each sample represents similar social strata within the society (young to middle-aged people, mostly university students, academic staff and members of the general public who were willing to ‘help the science’). The composition of the samples was therefore comparable across the sampled populations.

### 2.2. Rating sessions

Raters from each country assessed only stimuli from their own country. The ratings were self-paced and took part online (except for attractiveness of Cameroonian 2013 stimuli) using participants’ own electronic devices. Stimuli appeared on the screen in a pseudo-randomised order. The questionnaires were in English (Cameroon), Spanish (Colombia), Czech (Czechia), Farsi (Iran), and Turkish (Turkey). Raters were not compensated for their participation. Attractiveness was rated only by opposite-sex raters except for Turkey, where both male and female facial stimuli were rated by raters of both sexes (for alternative analyses with the subset of opposite-sex raters, see Table S4 and Figure S5 in the Supplementary Material). Perceived masculinity and femininity in the Cameroonian, Colombian, and Turkish sample were rated by raters of both sexes, while in Czechia and Iran, perceived masculinity and femininity were rated only by persons of the opposite sex. The number of stimuli in each country is reported in Table 1. The number of raters in each category and associated descriptive statistics are summarised in Table 2 and in Table S1 in the Supplementary Material. A detailed description of the rating process is in the Supplementary Material (Section S2.2, ‘Acquisition and processing of ratings’).

**Table 2.** Descriptive statistics of the rater sample

Sample	Rated attribute	No. of raters	Raters’ sex	Raters’ age (mean ± SD)	Raters’ weight	Raters’ height
Cameroon 2013	Women attractiveness	34	Men	22.21 ± 3.14	NA	NA
	Men attractiveness	28	Women	22.11 ± 3.89	NA	NA
	Masculinity/femininity^a^	77	Men and women	24.00 ± 4.12	66.62 ± 11.23	165.10 ± 7.75
Cameroon 2016	Women attractiveness	49	Men	22.96 ± 3.23	68.84 ± 8.73	169.70 ± 8.35
	Men attractiveness	51	Women	23.37 ± 4.25	65.18 ± 11.39	161.10 ± 5.32
	Masculinity/Femininity^a^	94	Men and women	22.99 ± 3.00	64.82 ± 9.53	164.50 ± 7.76
Colombia	Women attractiveness	432	Men	22.01 ± 3.92	NA	NA
	Men attractiveness	565	Women	21.85 ± 4.81	NA	NA
	Women femininity	432	Men	22.01 ± 3.92	NA	NA
	Men masculinity	565	Women	21.85 ± 4.81	NA	NA
Czechia	Women attractiveness	33	Men	28.18 ± 4.21	NA	182.50 ± 6.35
	Men attractiveness	89	Women	27.56 ± 4.23	NA	169.00 ± 5.50
	Women femininity	44	Men	33.00 ± 9.68	86.23 ± 12.23	182.40 ± 7.67
	Men masculinity	231	Women	32.04 ± 7.59	67.74 ± 15.32	168.30 ± 6.46
Iran	Women attractiveness	46	Men	37.30 ± 12.29	85.61 ± 15.13	178.60 ± 7.93
	Men attractiveness	41	Women	34.88 ± 9.91	65.73 ± 14.98	165.60 ± 6.07
	Women femininity	33	Men	27.73 ± 3.77	78.52 ± 12.93	178.30 ± 5.99
	Men masculinity	31	Women	29.35 ± 4.54	59.68 ± 9.20	164.60 ± 6.66
Turkey	Women attractiveness	1207^b^	862 women; 225 men; 120 unreported	All, 22.09 ± 3.66; women, 21.71 ± 2.80; men, 23.53 ± 5.69	All, 61.6 ± 12.33; women, 58.02 ± 9.66; men, 75.23 ± 11.90	All, 167.90 ± 7.79; women, 165.4 ± 7.79; men, 177.70 ± 6.09
	Men attractiveness					
	Masculinity/Femininity					

a In the Cameroonian and Turkish samples, perceived sex-typicality (masculinity of men, femininity of women) was rated by a combined set of male and female raters.

b Some Turkish raters did not report their attributes (age, sex, weight and height). For alternative analyses without those raters and split by raters’ sex, see Table S4 and Figure S5 in the online Supplementary Material.

Cameroonian stimuli from 2013 and 2016 were rated in two separate runs. Of all the datasets, only the perceived attractiveness of Cameroonian stimuli collected in 2013 was not rated using Qualtrics. It was rated in an offline purpose-made ‘ImageRater’ program visually similar to the Qualtrics interface. Cameroonian raters assessed the stimuli on a verbally anchored seven-point Likert scale (1, not at all attractive/masculine/feminine; 7, very attractive/masculine/feminine face). Each rater rated all the stimuli within a given set. Raters were mostly university students who resided in towns and villages of western Cameroonian provinces.

In Colombia, raters were recruited by JDL and his co-workers. They were mostly university students from Bogota, D.C. The sample consisted of 997 raters (565 women). They rated only a randomly chosen opposite-sex subset (*N* = 20) of the stimuli. The rating scale ranged from 1.0 to 10.0 (with one decimal place). The endpoints were also anchored verbally, with 1.0 not at all attractive/masculine/feminine and 10.0 very attractive/masculine/feminine face. All participants identified themselves as heterosexual.

Czech raters were recruited by fliers in university buildings, asked face to face or by email by two of the authors (VF, KK) and KK's other co-workers. The link was also shared via social networks through groups of Charles University students and academic staff. Only raters who identified themselves as heterosexual and completed the whole questionnaire were entered into the analysis.

Iranian raters (see Section S.2.2.4 in the online Supplementary Material) were recruited via email with a link to the questionnaires sent by one of the authors (FP), who also translated the original questionnaires from English into Farsi. Raters who identified themselves as homosexual, did not complete the entire questionnaire or rated all stimuli identically were excluded from subsequent analyses.

In Turkey, the rating data were collected online by one of the authors (AS). Participants were asked to rate a subset of the Turkish face database (Bogazici Face Database; see Saribay et al., 2018). Students from the Bogazici University were deliberately excluded from the pool of potential raters owing to potential bias stemming from familiarity with the stimuli. Raters saw only a subset of stimuli (*N* = 8 faces per rated attribute) in pseudorandomised order. They rated both the male and female stimuli.

### 2.3. Facial measures

#### 2.3.1. Skin lightness

In the Cameroonian (2013), Colombian, Iranian and Turkish targets, we measured skin lightness from facial photographs using ImageJ software (Schneider et al., 2012) with the plugin Color Transformer 2. In the Cameroon 2016 and Czech sample, facial skin lightness was measured *in vivo* with a spectrophotometer (Ocean Optics Flame-S, 200–850 nm, optical resolution 2 nm). All measurements were taken on three patches of the target face (forehead, left and right cheek) and expressed as the L* dimension of CIELab colour space (Hunter, 1958). According to Coetzee et al. (2012), such difference in the lightness measurement (*in vivo* by spectrophotometer vs. using facial photographs) should not affect the results. Nevertheless, we also ran the analysis with L* measured from photos in ImageJ in samples where lightness was originally based on *in vivo* spectrophotometric measurements. Results for these analyses are available in the Supplementary Material (Table S5 and Figure S6). The results were not substantially affected by the method of skin lightness measurement.

#### 2.3.2. Relative facial width

We measured bizygomatic facial width and upper facial height from the glabella to the border of the upper lip from facial photographs of the stimuli persons (Třebický et al., 2015). Then we calculated the fWHR as facial width divided by facial height (see also Section S2.4.2 in the Supplementary Material for a detailed description of the measurement).

#### 2.3.3. Facial shape analysis

We manually landmarked each facial photograph with 72 landmarks (36 landmarks and 36 semilandmarks) in tpsDig2 software, version 2.31 (Rohlf, 2015). We followed the definitions of standard landmarks positions previously used by Kleisner et al. (2019). Procrustes superimposition of all landmark configurations was done using the ‘gpagen()’ function in the R package Geomorph (Adams & Otárola-Castillo, 2013). Semilandmark positions were optimised based on minimising the Procrustes distances between corresponding points. For a more detailed description of facial shape analysis, see Section S.2.4.4 of the Supplementary Material.

We computed facial morphological averageness as each face's distance from the average facial configuration of its bearer's sex and population sample (e.g. averageness of Turkish male targets). The higher the value, the less average the facial configuration.

Next, we computed the level of facial sexual shape dimorphism (morphological sexual dimorphism) by projecting each facial configuration on a vector connecting the male and female average within a given sample (e.g. Turkish male and female targets – the scale uses both sexes within a given population sample). Higher negative values of sexual shape dimorphism indicate a more female-like facial shape, while higher positive values indicate a more male-like facial shape.

Note that all Iranian women in our sample wore the hijab. To compute the averageness of Iranian women and sexual shape dimorphism of Iranian men and women, we used the configuration of 49 innermost facial landmarks. Computations of morphological facial averageness and sexual shape dimorphism in the remaining groups were always based on all 72 landmarks.

### 2.4. Statistical analysis

All analyses were conducted using the R software for statistical computing (version 3.6.0; R Core Team, 2020). All datasets and R script are available at https://osf.io/va8pg/?view_only=021e11321855463f82fc64e6ceb5716a.

To assess interrater agreement on perceived attractiveness and sex-typicality, we computed Intraclass correlations using the ‘ICC()’ function of the ‘psych’ package (Revelle, 2018). We ran two-way mixed average score Intraclass correlations (3,*k*) for the Cameroonian, Czech and Iranian datasets where raters saw all targets. Raters in Colombia and Turkey saw only a subsample of the relevant targets, which is why for these two samples, we ran one-way random, average score Intraclass correlations (1,*k*) (Shrout & Fleiss, 1979). All intraclass correlations were very high (ICC > 0.9, see Table 1). For subsequent analyses, we calculated mean perceived attractiveness and perceived sex-typicality ratings for each photographed person.

**Figure 1.**
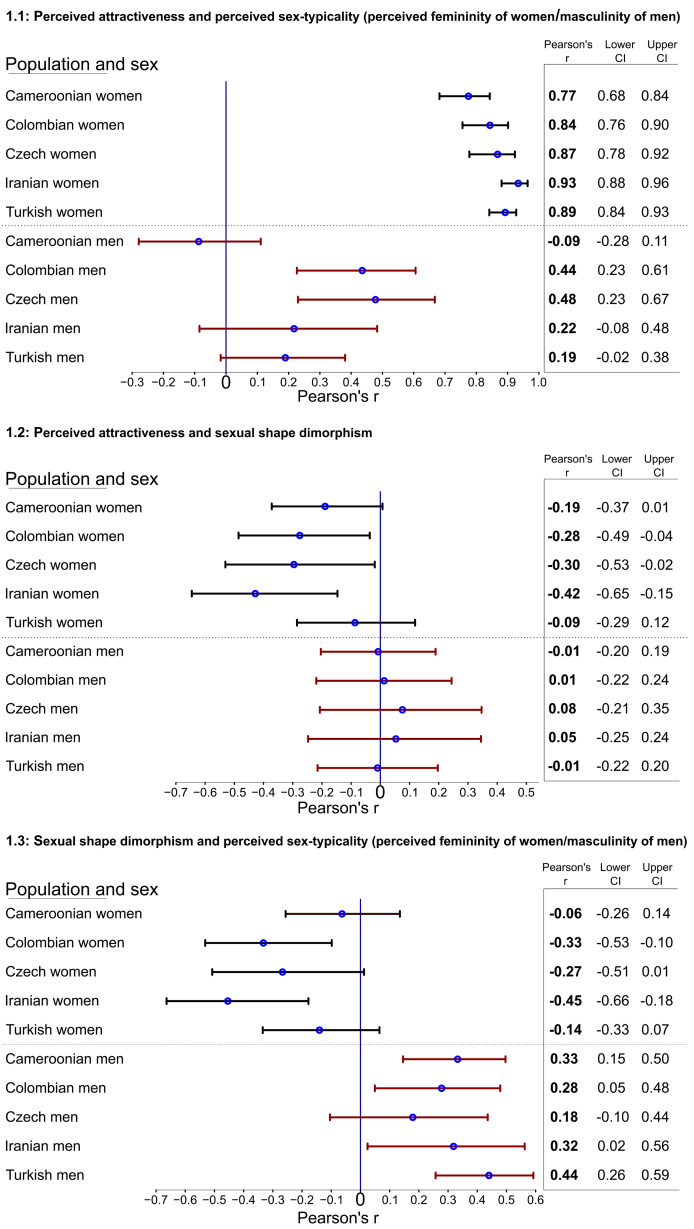
Forest plots displaying the relative strength of Pearson's correlations between perceived attractiveness and perceived sex-typicality (a), perceived attractiveness and sexual shape dimorphism (b) and between sexual shape dimorphism and perceived sex-typicality (c), with confidence intervals of each coefficient. Each row corresponds to a single sample (women from all five samples, men from all five samples, with sampled countries in alphabetical order). Blue circles represent Pearson's correlation coefficients (mean estimate on the given sample), while black lines stand for error bars defined as 95% confidence intervals around each mean correlation. A vertical line in zero (‘0’) enables us to inspect whether the confidence interval for a given correlation contains zero. The columns on the right side of the diagrams show coefficients of the associations. This figure facilitates a comparison of bivariate associations among the population samples.

Two-tailed Pearson's correlation coefficients (and their 95% confidence intervals) were used to investigate bivariate associations between all collected variables. The resulting *p*-values were adjusted for multiple comparisons using the Benjamini–Hochberg correction procedure. Unlike the Bonferroni correction, which controls for familywise error rate, Benjamini–Hochberg correction controls for the predicted (expected) proportion of errors among rejected null hypotheses, that is, the false discovery rate (Benjamini & Hochberg, 1995). It is therefore better suited for a non-confirmatory analysis where there is no dependence between multiple comparisons. The results of performed correlations and descriptive statistics (mean, SD, and range) for all variables are presented in Tables S2–S5 in the Supplementary Material. The associations were further visualised using heatmaps of Pearson correlation coefficients (see Figure S4 in Supplementary Material) using the ‘col_fun()’ function of the circlize package (Gu et al., 2014) and the ‘Heatmap()’ function of the ComplexHeatmap package (Gu et al., 2016) in R software, and subsequently edited in InkScape version 0.92.4. To visualise the strength of associations between measures of sexual dimorphism (SShD and perceived sex-typicality) and perceived attractiveness across the five samples, we created a forest plot (see Figure 1) using the ‘forestplot()’ function of the forestplot package (Gordon & Lumley, 2020).

**Figure 2.**
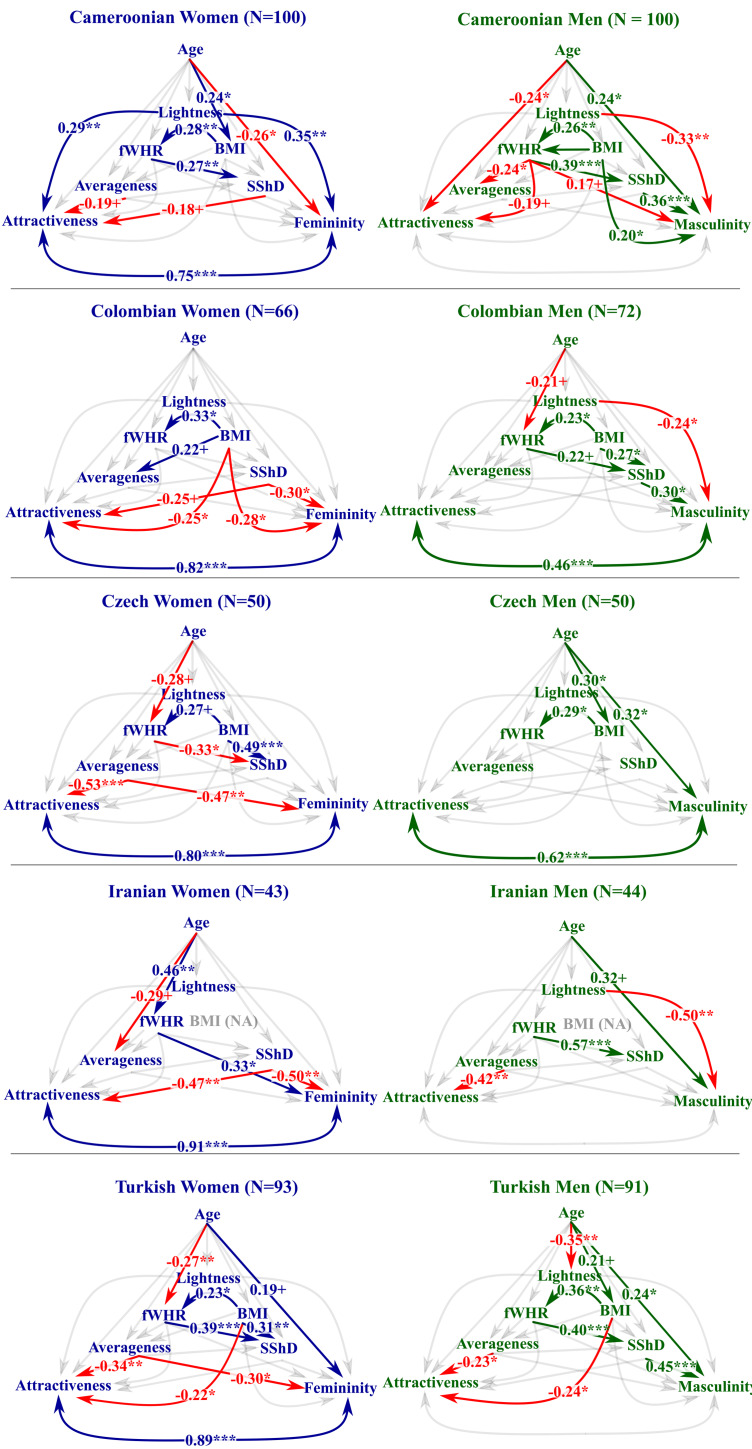
A visualisation of path analyses (multiple regression models) between the rated facial attributes (perceived sex-typicality and attractiveness) and facial measures ordered by the sex of the stimuli. Arrows represent the direction of the association. Non-significant paths are omitted. Association between perceived sex-typicality and attractiveness was treated as a correlation (i.e. the direction was not specified). Numbers next to the paths indicate the estimate of regression or correlation coefficient in a corresponding model with standardised variables. Red colour denotes a negative coefficient. The graph shows to what extent is the observed within-sample variability of each variable explained by other variable(s). In every sample, perceived femininity and attractiveness are closely mutually associated in the women's samples. In most population samples, perceived masculinity was not associated with perceived men's attractiveness. The significant paths mostly replicate significant Pearson's correlations (see Figure S4) (+ *p* < 0.10, *p < 0.05, ** *p* < 0.01, *** *p* < 0.001).

To explore causal relationships between age, skin lightness, fWHR, BMI, averageness, morphological sexual shape dimorphism, perceived sex-typicality and perceived attractiveness, we ran path analyses with ‘sem()’ function from the lavaan package for R (Rosseel, 2012). We conducted a separate path analysis for each stimulus sex/sample combination (e.g. Iranian female stimuli). Perceived sex-typicality and attractiveness were set as dependent variables (see Figure 2), while directionality of mutual interdependence between the two was not decided and we treated it as a correlation; for a detailed description of the model development, see Section S.2.5 in the Supplementary Material. Paths designated in this study were based on evidence from literature, formal logic and our decisions (see also Kane & Ashbaugh, 2017). The aim of the study was to explore the proposed paths, not to confirm a pattern as an objective causality. Because the number of observations per estimated parameter was relatively low, robust *p*-values were obtained using a permutation test with 10,000 iterations, where the full models were estimated on randomised datasets.

## 3. Results

### 3.1. Correlational analyses

In women, we found a significant positive correlation between perceived attractiveness and perceived femininity in all five samples: Cameroonian, *r*(98) = 0.77; 95% CI [0.68, 0.84], *p* < 0.001; Colombian, *r*(64) = 0.84 [0.76, 0.90], *p* < 0.001; Czech, *r*(48) = 0.87 [0.78, 0.92], *p* < 0.001; Iranian, *r*(41) = 0.93 [0.88, 0.96], *p* < 0.001; and Turkish, *r*(92) = 0.89 [0.84, 0.93], *p* < 0.001. Furthermore, in the sample of Cameroonian women, both perceived attractiveness and perceived femininity were significantly and positively correlated with skin lightness (*r*(98) = 0.31 [0.12, 0.47], *p* = 0.014; and *r*(98) = 0.36 [0.17, 0.52], *p* = 0.004, respectively). In the rest of women's samples, the correlation between skin lightness and perceived femininity or attractiveness was not significant (*p* > 0.05, *p*-value after Benjamini–Hochberg correction; see Figure S4).

In men, perceived masculinity and attractiveness were significantly and positively correlated only in the Colombian and Czech sample (*r*(70) = 0.44 [0.23, 0.61], *p* = 0.004; *r*(48) = 0.48 [0.23, 0.67], *p* = 0.013, respectively). In samples of the Cameroonian, Iranian and Turkish male faces, perceived masculinity was significantly negatively associated with facial skin lightness (*r*(98) = −0.38 [−0.54, −0.20], *p* = 0.001; *r*(42) = −0.63 [−0.78, −0.40], *p* < 0.001; *r*(89) = 0.32 [−0.49, −0.12], *p* = 0.009, respectively), meaning that darker men were perceived as more masculine.

Morphological averageness was significantly correlated with perceived attractiveness in Iranian men (*r*(42) = −0.41 [−0.63, −0.13], *p* = 0.028), with perceived femininity (*r*(48) = −0.44 [−0.64, −0.19], *p* = 0.011) and attractiveness (*r*(48) = −0.52 [−0.70, −0.28], *p* = 0.002) in Czech women, and with perceived femininity (*r*(92) = −0.29 [−0.46, −0.09], *p* = 0.03) and attractiveness (*r*(92) = −0.34 [−0.51, −0.14], *p* = 0.007) in Turkish women. In these samples, more average faces (i.e. ‘averageness’ values closer to zero) were thus perceived as more sex-typical and/or attractive.

Finally, sexual shape dimorphism was significantly correlated with perceived masculinity in the sample of Cameroonian men (*r*(98) = 0.33 [0.15, 0.50], *p* = 0.005), meaning that men with more male-like facial shape were perceived as more masculine. Sexual shape dimorphism also correlated with perceived femininity in Colombian women (*r*(64) = −0.33 [−0.53, −0.10], *p* = 0.045), implying that women with more female-like facial shape were perceived as more feminine, with perceived femininity (*r*(41) = −0.45 [−0.66, −0.18], *p* = 0.016) and attractiveness (*r*(41) = −0.43 [−0.65, −0.15], *p* = 0.022) in Iranian women, and with perceived masculinity of Turkish men (*r*(89) = 0.44 [0.26, 0.59], *p* < 0.001). In the rest of the samples, the association between sexual shape dimorphism and perceived sex-typicality was not significant after Benjamini–Hochberg correction (see Figure S4). Figure 1 presents forest plots for correlations between perceived sex-typicality, sexual shape dimorphism and perceived attractiveness for each population sample and sex. All correlation coefficients are reported in detail in Figure S4 and Table S2–S5 in the Supplementary Material.

### 3.2. Path analyses

#### 3.2.1. Women's faces

Significant paths among variables mostly replicated the significant correlations reported above. In all women's samples, there were significant positive residual correlations between perceived femininity and attractiveness (*r* = 0.75; 95% CI [0.56, 0.94] for Cameroonian, 0.82 [0.57, 1.00] for Colombian, 0.80 [0.59, 1.00] for Czech, 0.91 [0.65, 1.00] for Iranian and 0.89 [0.66, 1.00] for Turkish women, *p* < 0.001 in all cases).

In Cameroonian women, there was a significant positive association (partial regression) between facial skin lightness and both perceived femininity and attractiveness (*β* = 0.35 [0.18, 0.52], *p* = 0.001; *β* = 0.29 [0.11, 0.47], *p* = 0.004, respectively).

Concerning correlations between facial morphology and perceived traits, we found a significant association between sexual shape dimorphism and perceived femininity in the Colombian and Iranian women's sample (*β* = −0.30 [−0.53, −0.07], *p* = 0.019; *β* = −0.50 [−0.74, −0.25], *p* = 0.001, respectively), while in the Iranian women's sample, sexual shape dimorphism was significantly associated with perceived attractiveness (*β* = −0.47 [−0.71, −0.22], *p* = 0.002). More feminine facial morphology had a more negative value of sexual shape dimorphism, which indicates that feminine facial shape was perceived as more attractive in Iran and as more feminine in the Iranian and Colombian women's sample. In Colombia, partial regression between sexual shape dimorphism and perceived attractiveness was not significant (as noted above, we report the ‘robust *p*-values’ based on bootstrapping) but was of a similar magnitude and direction (*β* = −0.25 [−0.49, −0.02], *p* = 0.051). In the Czech women's sample, morphological facial averageness was significantly associated with both perceived femininity and attractiveness (more average faces were perceived as more feminine and attractive, *β* = −0.47 [−0.70, −0.25], *p* = 0.001; *β* = −0.53 [−0.74, −0.33], *p* < 0.001, respectively). The same held true for the Turkish female sample (*β* = −0.30 [−0.49, −0.11], *p* = 0.002 for the association between morphological averageness and perceived femininity; *β* = −0.34 [−0.53, −0.15], *p* < 0.001 between morphological averageness and perceived attractiveness).

Perceived facial attractiveness and perceived femininity in women's samples were also related to other variables: in Colombia, women with a higher BMI were perceived as significantly less feminine (*β* = −0.28 [−0.52, −0.05], *p* = 0.026) and less attractive (*β* = −0.25 [−0.50, −0.01], *p* = 0.041), while in the Cameroonian women's sample, age was significantly negatively associated with perceived femininity (*β* = −0.26 [−0.44, −0.08], *p* = 0.017).

Concerning associations between the predictors themselves, in Cameroonian (*β* = 0.28 [0.08, 0.47], *p* = 0.005), Colombian (*β* = 0.33 [0.10, 0.56], *p* = 0.010) and Turkish women's samples (*β* = 0.23 [0.04, 0.42], *p* = 0.029), fWHR was significantly positively associated with BMI. In Cameroon (*β* = 0.27 [0.08, 0.46], *p* = 0.008) and Turkey (*β* = 0.39 [0.21, 0.58], *p* < 0.001), women with a higher fWHR exhibited lower levels of female-like sexual shape dimorphism, while in Czechia, women with higher fWHR exhibited higher levels of female-like SShD (*β* = −0.33 [−0.59, −0.07], *p* = 0.025). Age was significantly associated with BMI (*β* = 0.24 [0.05, 0.43], *p* = 0.025) only in the Cameroonian women's sample and with fWHR in the Iranian (*β* = 0.46 [0.19, 0.72], *p* = 0.002) and Turkish women's sample (*β* = −0.27 [−0.46, −0.08], *p* = 0.007).

#### 3.2.2. Men's faces

Perceived masculinity of Colombian and Czech male faces was significantly positively correlated with their perceived attractiveness (*r* = 0.46 [0.24, 0.67], *p* < 0.001 and *r* = 0.62 [0.36, 0.88], *p* < 0.001, respectively). In samples from the other populations, the association between perceived attractiveness and perceived masculinity was not significant (*r* = −0.07 [−0.22, 0.07], *p* = 0.57; *r* = 0.16 [−0.02, 0.33], *p* = 0.53; and *r* = 0.19 [0.03, 0.36], *p* = 0.14 for Cameroonian, Iranian and Turkish male faces, respectively). Facial skin lightness was significantly negatively associated with perceived masculinity in the Cameroonian (*β* = −0.33 [−0.49, −0.18], *p* = 0.001), Colombian (*β* = −0.24 [−0.44, −0.03], *p* = 0.041) and Iranian (*β* = −0.50 [−0.70, −0.30], *p* = 0.001) male samples.

Concerning morphological and perceived facial traits, the following significant associations were observed: in the Cameroonian (*β* = 0.36 [0.19, 0.54], *p* < 0.001), Colombian (*β* = 0.30 [0.07, 0.53], *p* = 0.014) and Turkish sample (*β* = 0.45 [0.24, 0.66], *p* < 0.001) more male-like facial shapes (sexual shape dimorphism) were perceived as more masculine (perceived masculinity). In the Iranian (*β* = −0.42 [−0.68, −0.15], *p* = 0.007) and Turkish samples (*β* = −0.23 [−0.43, −0.04], *p* = 0.024), facial configurations closer to the average (morphological averageness) were perceived as more attractive.

Body mass index was significantly positively associated with fWHR in Cameroonian (*β* = 0.26 [0.07, 0.45], *p* = 0.009), Colombian (*β* = 0.23 [0.01, 0.45], *p* = 0.048), Czech (*β* = 0.29 [0.01, 0.57], *p* = 0.041) and Turkish (*β* = 0.36 [0.16, 0.55], *p* < 0.001) men, meaning that relatively heavier men had relatively wider faces (in the Iranian stimuli, we did not measure weight and height and thus could not compute this association). In Cameroonian men, fWHR was significantly associated with morphological averageness (*β* = −0.24 [−0.43, −0.04], *p* = 0.019) and sexual shape dimorphism (*β* = 0.39 [0.21, 0.58], *p* < 0.001). The fWHR was also significantly positively related to sexual shape dimorphism in Iranian (*β* = 0.57 [0.33, 0.81], *p* < 0.001) and Turkish men (*β* = 0.40 [0.22, 0.59], *p* < 0.001).

See also Figures S5 and S6 in the Supplementary Material for path analyses with alternative variables and datasets. Table S6 in the Supplementary Material reports the full ‘lavaan’ output for all fitted path analyses.

## 4. Discussion

In this study, we investigated the associations between perceived attractiveness, perceived sex-typicality and facial sexual shape dimorphism (morphological sexual dimorphism) in samples from five distant populations, namely Cameroon, Colombia, Czechia, Iran and Turkey. We also explored whether these associations were affected by other factors (morphological facial averageness, skin lightness, relative facial width, body mass and age).

As predicted (see Table 3), raters strongly preferred women who were perceived as more feminine in all samples. On the other hand, raters did not agree on the preferred degree of perceived male sex-typicality. In fact, only raters from the Czech and Colombian samples preferred men who were perceived as more masculine. Morphometric variables (facial morphological averageness, sexual shape dimorphism), relative facial width and measured skin lightness were inconsistent predictors of perceived scales: they predicted perceived characteristics only in a subset of the samples.

**Table 3.** Outline of predictions (a) and significant results (b)

(a) Predictions				
Variable ↓	Women		Men	
Perceived sex-typicality	Feminine faces perceived more attractive		Masculine faces perceived more attractive	
Sexual shape dimorphism (SShD)	Female-typical SShD perceived as more *feminine* and attractive		Male-typical SShD perceived as more *masculine* and attractive	
Morphological averageness	More average facial configurations perceived as more attractive		More average facial configurations perceived as more attractive	
Facial skin lightness^1^	Lighter faces perceived as more attractive and feminine		Darker faces perceived as more masculine	
Relative facial width (fWHR)	Wider faces perceived as less feminine		Wider faces perceived as more masculine (Geniole et al. 2015)	
Relative weight (BMI)^2^	Heavier stimuli perceived as less attractive		Heavier stimuli perceived as less attractive	
Age^2^	Younger stimuli perceived as more attractive and feminine		Younger stimuli perceived as more attractive	
(b) Results based on path analyses, with non-significant trends with *p*-value [>0.05, <0.1] omitted				
Variable ↓	Women	(Attr|Fem)^3^	Men	(Attr|Masc)^3^
Perceived sex-typicality	Femininity perceived as more attractive across samples	5|NA	Masculinity perceived as more attractive in two samples	2|NA
Sexual Shape Dimorphism	Female-typical SShD perceived as more attractive in one, more feminine in two samples	1|2	Male-typical SShD perceived as more masculine in three samples	0|3
Morphological averageness	More average faces perceived as more feminine and attractive in two samples	2|2	More average faces perceived as more attractive in two samples	2|0
Facial skin lightness	Lighter women perceived as more attractive and feminine in one sample	1|1	Darker men perceived as more masculine in three samples	0|3
Relative facial width (fWHR)	No effect	0|0	Non-significant trends	0|0
Relative weight (BMI)^2^	Lower BMI perceived as more attractive in two samples, more feminine in one sample	2|1	Higher BMI perceived as more masculine in one sample, less attractive in one sample	1|1
Age^2^	Younger women perceived as more feminine in one sample	0|1	Younger men perceived as more attractive in one, older men perceived as more masculine in three samples	1|3

1 Predictions within a given sample (e.g. darker/lighter within an Iranian male sample).

2 Predictions and results based on age and BMI are not further discussed because they go beyond the scope of the current study. For a more detailed review and anticipated effects of variables which were not discussed in the Introduction, see Sections S1 and S2.5 in Supplementary Material.

3 Significant result for Attractiveness|Perceived Sex-typicality; *N* out of five female and five male samples, from four samples for each sex for BMI.

### 4.1. Association between perceived sex-typicality and perceived attractiveness

The hypothesis according to which preferred visual facial traits should be invariant across samples from distant populations (Langlois et al., 2000) found support in our results only in part. In all five female samples, perceived attractiveness and perceived women's femininity were positively associated. Concerning preference for femininity, our results thus converge with previous evidence to the effect that perceived female femininity is preferred across populations (Little et al., 2011), except for cases where the sampled populations are distant, visually distinctive and/or inhabit various environments (cf. Marcinkowska et al., 2014).

Such high correlation between perceived femininity and attractiveness conforms to the evolutionary model and its implicit assumptions. It has been proposed, mostly based on indirect evidence, that femininity in women's faces presents an honest cue to proper hormone-based development (Probst et al., 2016; Thornhill & Grammer, 1999), fertility and reproductive health (Law Smith et al., 2006) and should be therefore preferred. Moreover, our results across samples replicate findings of recent studies which identified a strong association between facial femininity and attractiveness using various research design (Foo, Simmons et al., 2017; Mogilski & Welling, 2017; Muñoz-Reyes et al., 2015; Smith et al., 2009). Contrary to this assumption of universal preference of female femininity, other scholars suggested that in harsh environments (De Barra et al., 2013; Penton-Voak et al., 2004) and small rural populations (Scott et al., 2014), preference for female sex-typicality should be weaker or absent altogether. In our study, however, the magnitude of femininity preference was relatively stable across the samples from distant populations. Accordingly, female femininity, eventually pointing to sexual maturity and reproductive health, may present a women's characteristic that is universally preferred.

For men, we observed a substantial variation across samples in the magnitude of association between perceived masculinity and attractiveness. The results therefore do not support unequivocal conclusions that either masculine (Foo, Simmons, et al., 2017; Skrinda et al., 2014) or relatively more feminine (Perrett et al., 1998; Rhodes et al., 2000) facial traits are universally preferred in male faces. Perceived masculinity was considered attractive only in two of the samples, the Czech and the Colombian one. In the Iranian and Turkish sample, the association was also positive but not statistically significant.

Preference for masculinity might be the result of different adaptive processes in Czech and Colombian society but it is also possible that the results converge owing to similarities between our Czech and Colombian sample (not the whole populations). In two studies, Borras-Guevara et al. (2017a, b) found a negative association between Colombian women's masculinity preference in manipulated male faces and Colombian women's fear of domestic violence. Importantly, raters in these studies represented various social strata of the Colombian society. Our targets and raters, on the other hand, were for the most part university students from Bogota. It is thus likely that they represented a relatively affluent social group where preference for masculine male partners is not counteracted by fear of domestic violence. The issue of within-populations variance of preferences could, however, be addressed only in a study that would measure and compare women's relative safety from male violence, wealth distribution, and the perception of these environmental factors in both the Czech and Colombian societies.

Women's preferences in Middle Eastern countries in our sample (Iran and Turkey) were predicted neither by sexual shape dimorphism nor by perceived sex-typicality. Both Iranian and Turkish female raters preferred morphological averageness of facial shape in the male stimuli.

Future studies should explore this observed absence of preference for sex-typicality in Middle East countries with regard to locally important cultural factors. In particular, it should be taken into account that in Middle Eastern countries, the tradition of arranged marriages is still widespread, as is women's subordinate social role and strong interpersonal bonds (Cindoglu et al., 2011; Friedland et al., 2016). Parents and other relatives who negotiate and arrange a marriage may not perceive sex-typicality as highly important for their choice. Nonetheless, recent evidence shows that people in both countries choose their partners substantially more freely than in the past (Hart, 2007; Honarvar et al., 2016; Atari & Jamali, 2016a, b), which might imply that the preference for average facial configurations displayed by our local raters could mirror local adaptations in actual mate choice, unbiased by arranged marriages.

### 4.2. Association between shape variables and perceived characteristics

Previous studies revealed a weak to moderate association between perceived and morphological sexual dimorphism (Komori et al., 2011; Mitteroecker et al., 2015). In the current study, neither perceived femininity nor perceived masculinity and attractiveness were associated with sexual shape dimorphism (morphological sexual dimorphism) universally across all samples. When restricted only to significant effects in path analyses, more female-like shape indicated more attractive ratings only in one of the five women's samples (Iranian). In two populations (Colombian and Iranian), women with more female-like facial shape were also perceived as more feminine. In men, a more male-like facial configuration was predictive of higher perceived masculinity in three of the five samples (Cameroonian, Colombian, and Turkish). All in all, we thus found no universal association between perceived and measured sex-typicality in either sex, although in some of the samples, the association ran in the predicted direction.

Facial morphological averageness did not predict perceived characteristics consistently either. In three of the women's samples (Czech, Iranian and Turkish) and one men's sample (Turkish), more average facial configurations were perceived as more attractive. In the Czech and Turkish women sample, more average facial configurations were perceived as more feminine. As suggested by current research, it is possible that facial averageness is a trait that is relatively less important for the perception of facial characteristics than previously thought (Foo, Simmons, et al., 2017; Holzleitner et al., 2019; Jones & Jaeger, 2019). It seems, therefore, that morphological variables beyond actual dimorphism are not good predictors of the perceived facial attractiveness and perceived sex-typicality.

Measured morphometric variables used in this study express facial shape variance as a single number. Human perception is not, however, a computational device that processes shape in that way. As suggested by plastic surgery, cosmetics, and related fields, some parameters of facial shape are more important for perceived attractiveness than others. Such features include relative lip size, lower face size (Penna et al., 2015) and eyebrow size and shape (Schreiber et al., 2005). Moreover, preferred traits may be a combination of mature, neotenous and expressive traits (Borelli & Bernerburg, 2010; Cunningham et al., 1990), not just juvenile/submissive traits (in women) and mature/dominant traits (in men). Taken together, it is unlikely that the complex phenomenon of human facial trait assessment could be fully captured by a single morphometric measure.

### 4.3. Sexual dimorphism in the fWHR

The fWHR was positively associated with sexual shape dimorphism in Cameroonian and Turkish faces of both sexes and in Czech female and Iranian male faces. Such positive association implies that Cameroonian and Turkish men and women with more masculine facial configurations had relatively wider faces. Past studies identified a slight sexual dimorphism in fWHR (with men having relatively wider faces; Carré & McCormick, 2008), but a more recent study cast doubt on sexual dimorphism in fWHR when it ran analyses that controlled for sexual dimorphism in body size (Kramer, 2015). Özener (2012) found no sexual dimorphism in fWHR in a Turkish university student sample. Although fWHR is no longer considered a sexually dimorphic measure, our data suggest that fWHR is associated with more male sex-typical facial shape at least in some of our samples. Except for the sample of Czech women, fWHR was also significantly positively associated with BMI in all samples for which BMI was available. In short, it thus turned out that relatively heavier people also have wider faces regardless of their sex.

### 4.4. Association between skin lightness and perceived characteristics

We further predicted that skin lightness, which has been associated with perceived sex-typicality and healthiness in previous research (Carrito & Semin, 2019; Stephen & Perrett, 2016), should be positively related to perceived attractiveness and sex-typicality (with darker men being perceived as more masculine) in all the five population samples. In the samples of Cameroonian, Colombian and Iranian men, faces with a darker skin were perceived as more masculine. In the Czech and Turkish men's sample, facial skin lightness was not associated with perceived masculinity but the statistically non-significant associations for both Czech (*β* = −0.15) and Turkish (*β* = −0.13) men were in the same direction as in the rest of the samples. Owing to a low statistical power of our study, we cannot decide whether this association is ecologically irrelevant in these two countries or whether there indeed exists a stable association between darker facial skin and perceived sex-typicality across populations.

Regarding women, skin lightness was significantly associated with perceived facial characteristics only in the Cameroonian female sample. Raters perceived Cameroonian women with a lighter complexion as being both more feminine (perceived femininity) and more attractive. This observation is in line with previous studies which identified facial skin colouration as a more important cue to the perception of facial attributes in African than in non-African populations (Coetzee et al., 2014; Kleisner et al., 2017; Strom et al., 2012).

### 4.5. Limitations of the study

The aim of the current study was to explore the association between perceived and measured facial traits in samples from five distant countries. To acquire comparable data and results from each investigated country, we tried to keep the methodology of data collection as similar as possible. Despite these efforts, we did not manage to maintain all photo acquisition parameters identical throughout (e.g. camera sensor size or camera-to-subject distance), nor were we able to secure identical lighting conditions in all samples. Within each sample, however, photo acquisition standards were identical and maintained.

The questionnaire for Colombian raters differed in the granularity of the rating scale: in Colombia, the scale ranged from 1.0 to 10.0 with one decimal place, while elsewhere raters responded using seven-point Likert scales. On the other hand, we analysed all data within-sample rather than aggregating them across the samples and report standardised coefficients. There is, therefore, no reason to suspect that scale variability affected our results.

Electronic devices used by raters may have caused some differences in ratings owing to, for instance, differences in the screen size (Třebický et al., 2018). Future studies should either control for the type of electronic device used or conduct rating sessions under controlled laboratory conditions.

On an individual level, preference for sexual dimorphism in men's faces may have been influenced by current fertility status and hormonal regulation (Jones et al., 2019 and citations therein) or raters’ relationship status (Little et al., 2002, 2007). Unfortunately, we did not collect these data about participating raters. Concerning other factors on a subpopulation scale, in the Turkish and Cameroonian samples we explored whether some raters’ attributes and some stimuli affected the ratings and significant paths (e.g. the method of skin lightness measurement, combining and separating subsamples, raters’ sex; see Table S4 and Figure S5 in the Supplementary Material). Unfortunately, in the Cameroonian, Czech and Iranian sample, only tens of individual ratings were available: the subsets they yielded are thus unlikely to form a reliable base for a comparison of stability of perception across various social and ethnic groups within the sampled populations.

Although we measured and controlled for several variables (age, BMI, fWHR) and checked for some aspects of raters’ identity, we certainly did not address all possible confounding variables. Other facial attributes, such as skin colouration with respect to redness and yellowness (Carrito et al., 2016), contrast between facial features and skin (Stephen & McKeeganh, 2010), or facial hair (Clarkson et al., 2020) might likewise affect perception of sex-typicality and attractiveness. Future studies should also account for cross-cultural differences in characteristics that people value in potential mates, local beliefs and customs, and for the prevailing type of spousal choice.

## 5. Conclusions

Current studies on the association between facial attractiveness and sexual dimorphism frequently use a combination of manipulated stimuli and a forced-choice paradigm that dichotomises participants’ decision-making. It is therefore appropriate to investigate whether conclusions yielded by these setups can be replicated using different methods. Our results, which were based on non-manipulated facial photographs, provide limited support to the hypothesis that raters prefer sex-typical features across populations from distant countries. While perceived femininity of women was preferred in all population samples included in our study and the strength of perceived attractiveness–femininity association was relatively stable, thus suggesting a potential universality of the association, men's perceived facial masculinity was preferred only in the Czech and Colombian sample, that is, in two distant populations. Our raters from urbanised Iranian and Turkish populations did not prefer facial masculinity in men and the same applied to raters from the relatively more rural Cameroonian society. Our study therefore points to a population-specific association of perceived male sex-typicality and attractiveness based on natural facial stimuli of both sexes.

Further, we showed that morphometric variables (sexual shape dimorphism and facial averageness) and measured skin lightness were only moderate and inconsistent predictors of perceived sex-typicality. Presumably owing to this weak predictive power, these variables also did not predict perceived attractiveness across the samples. Measured and perceived sex-typicality tell a different story with respect to human preference. Ideally, different terms should be applied to measured and perceived traits associated with sex-typicality and averageness and researchers ought to bear in mind that some traits (e.g. skin lightness) may be population-specific predictors of perceived attributes, in this case sex-typicality.

## Data Availability

Original data and other supplementary materials to this article are available online at https://osf.io/va8pg/?view_only=021e11321855463f82fc64e6ceb5716a.

## References

[ref1] Adams, D. C., & Otárola-Castillo, E. (2013). Geomorph: An R package for the collection and analysis of geometric morphometric shape data. Methods in Ecology and Evolution, 4(4), 393–399. 10.1111/2041-210X.12035

[ref2] Anzures, G., Quinn, P. C., Pascalis, O., Slater, A. M., Tanaka, J. W., & Lee, K. (2013). Developmental origins of the other-race effect. Current Directions in Psychological Science, 22(3), 173–178. 10.1177/0963721412474459 24049246 PMC3773883

[ref3] Aoki, K. (2002). Sexual selection as a cause of human skin colour variation: Darwin's hypothesis revisited. Annals of Human Biology, 29(6), 589–608. 10.1080/0301446021000019144 12573076

[ref4] Apicella, C. L., Little, A. C., & Marlowe, F. W. (2007). Facial averageness and attractiveness in an isolated population of hunter–gatherers. Perception, 36, 1813–1820. 10.1068/p5601 18283931

[ref5] Atari, M., & Jamali, R. (2016a). Dimensions of women's mate preferences: Validation of a mate preference scale in Iran. Evolutionary Psychology, 14(2), 1–10. 10.1177/1474704916651443

[ref6] Atari, M., & Jamali, R. (2016b). Mate preferences in young Iranian women: Cultural and individual difference correlates. Evolutionary Psychological Science, 2(4), 247–253. 10.1007/s40806-016-0060-x

[ref7] Badaruddoza (2007). A paradox of human mate preferences and natural selection. Journal of Human Ecology, 21(3), 195–197. 10.1080/09709274.2007.11905972

[ref8] Benjamini, Y., & Hochberg, Y. (1995). Controlling the false discovery rate: A practical and powerful approach to multiple testing. Journal of the Royal Statistical Society. Series B *(*Methodological*)*, 57(1), 289–300. https://cran.r-project.org/package=forestplot

[ref9] Booth, A., Dabbs, J. M., & Dabbs, J. M. Sr (1993). Testosterone and men's marriages. Social Forces, 72(2), 463–477.

[ref10] Boothroyd, Lynda G., Jones, B. C., Burt, D. M., & Perrett, D. I. (2007). Partner characteristics associated with masculinity, health and maturity in male faces. Personality and Individual Differences, 43(5), 1161–1173. 10.1016/j.paid.2007.03.008

[ref11] Boothroyd, Linda G., Jones, B. C., DeBruine, L. M., & Perrett, D. I. (2008). Facial correlates of sociosexuality. Evolution and Human Behavior, 29(3), 211–218. 10.1016/j.evolhumbehav.2007.12.009

[ref12] Borelli, C., & Berneburg, M. (2010). ‘Beauty lies in the eye of the beholder’? Aspects of beauty and attractiveness. JDDG – Journal of the German Society of Dermatology, 8(5), 326–330. 10.1111/j.1610-0387.2009.07318.x 20537001

[ref13] Borras-Guevara, M. L., Batres, C., & Perrett, D. I. (2017a). Aggressor or protector? Experiences and perceptions of violence predict preferences for masculinity. Evolution and Human Behavior, 38(4), 481–489. 10.1016/j.evolhumbehav.2017.03.004

[ref14] Borras-Guevara, M. L., Batres, C., & Perrett, D. I. (2017b). Domestic violence shapes Colombian women's partner choices. Behavioral Ecology and Sociobiology, 71(175), 13–14. 10.1007/s00265-017-2405-2 PMC5694761 29200603

[ref15] Bronstad, P. M., & Russell, R. (2007). Beauty is in the ‘we’ of the beholder: Greater agreement on facial attractiveness among close relations. Perception, 36(11), 1674–1681. 10.1068/p5793 18265847

[ref16] Brooks, R., Scott, I. M., Maklakov, A. A., Kasumovic, M. M., Clark, A. P., & Penton-Voak, I. S. (2011). National income inequality predicts women's preferences for masculinized faces better than health does. Proceedings of the Royal Society B: Biological Sciences, 278, 810–812. 10.1038/29772 PMC3049041 21147809

[ref17] Burke, D., Nolan, C., Hayward, W. G., Russell, R., & Sulikowski, D. (2013). Is there an own-race preference in attractiveness? Evolutionary Psychology, 11(4), 855–872.23948346

[ref18] Buss, D. M. (1989). Sex differences in human mate preferences: Evolutionary hypothesis tested in 37 cultures. Behavioral and Brain Sciences, 12, 1–49. 10.1017/S0140525X00023992

[ref19] Carre, J. M., & McCormick, C. M. (2008). In your face: Facial metrics predict aggressive behaviour in the laboratory and in varsity and professional hockey players. Proceedings of the Royal Society B: Biological Sciences, 275(1651), 2651–2656. 10.1098/rspb.2008.0873 PMC2570531 18713717

[ref20] Carrito, M. de L., Santos, I. M. B. dos, Lefevre, C. E., Whitehead, R. D., Silva, C. F. da, & Perrett, D. I. (2016). The role of sexually dimorphic skin colour and shape in attractiveness of male faces. Evolution and Human Behavior, 37(2), 125–133. 10.1016/j.evolhumbehav.2015.09.006

[ref21] Carrito, M. L., & Semin, G. R. (2019). When we don't know what we know – Sex and skin color. Cognition, 191(June), 103972. 10.1016/j.cognition.2019.05.009 31228668

[ref22] Cindoglu, D., Çemrek, M., Toktas, S., & Zencirci, G. (2011). The family in Turkey: The battleground of the modern and the traditional. In C. B. Hennon & S. M. Wilson (Eds.), Families in a global context (pp. 235–263). New York: Routledge. 10.4324/9780203836941

[ref23] Clarkson, T. R., Sidari, M. J., Sains, R., Alexander, M., Harrison, M., Mefodeva, V., … Dixson, B. J. W. (2020). A multivariate analysis of women's mating strategies and sexual selection on men's facial morphology. Royal Society Open Science, 7, 191209. 10.1098/rsos.191209 32218951 PMC7029899

[ref24] Coetzee, V., Faerber, S. J., Greeff, J. M., Lefevre, C. E., Re, D. E., & Perrett, D. I. (2012). African perceptions of female attractiveness. PLoS ONE, 7(10), 3–8. 10.1371/journal.pone.0048116 PMC3483252 23144734

[ref25] Coetzee, V., Greeff, J. M., Stephen, I. D., & Perrett, D. I. (2014). Cross-cultural agreement in facial attractiveness preferences: The role of ethnicity and gender. PLoS ONE, 9(7). 10.1371/journal.pone.0099629 PMC4079334 24988325

[ref26] Cunningham, M. R., Barbee, A. P., & Pike, C. L. (1990). What do women want? Facialmetric assessment of multiple motives in the perception of male facial physical attractiveness. Journal of Personality and Social Psychology, 59(1), 61–72. 10.1037/0022-3514.59.1.61 2213490

[ref27] De Barra, M., DeBruine, L. M., Jones, B. C., Mahmud, Z. H., & Curtis, V. A. (2013). Illness in childhood predicts face preferences in adulthood. Evolution and Human Behavior, 34(6), 384–389. 10.1016/j.evolhumbehav.2013.07.001

[ref28] DeBruine, L. M., Jones, B. C., Crawford, J. R., Welling, L. L. M., & Little, A. C. (2010). The health of a nation predicts their mate preferences: Cross-cultural variation in women's preferences for masculinized male faces. Proceedings. Biological Sciences/The Royal Society, 277(1692), 2405–2410. 10.1098/rspb.2009.2184 PMC2894896 20236978

[ref29] DeBruine, L. M., Jones, B. C., Little, A. C., Crawford, J. R., & Welling, L. L. M. (2011). Further evidence for regional variation in women's masculinity preferences. Proceedings of the Royal Society of London B: Biological Sciences, 278(1707), 813–814. 10.1098/rspb.2010.2200

[ref30] DeBruine, L. M., Jones, B. C., Smith, F. G., & Little, A. C. (2010). Are attractive men's faces masculine or feminine? The importance of controlling confounds in face stimuli. Journal of Experimental Psychology: Human Perception and Performance, 36(3), 751–758. 10.1037/a0016457 20515201

[ref31] Deffenbacher, K. A., Vetter, T., Johanson, J., & O'Toole, A. J. (1998). Facial aging, attractiveness, and distinctiveness. Perception, 27(10), 1233–1243. 10.1068/p271233 10505202

[ref32] Dixson, Barnaby J., Dixson, A. F., Bishop, P. J., & Parish, A. (2010). Human physique and sexual attractiveness in men and women: A New Zealand–U.S. *comparative study.* Archives of Sexual Behavior, 39(3), 798–806. 10.1007/s10508-008-9441-y 19139985

[ref33] Dixson, Barnaby J., Little, A. C., Dixson, H. G., & Brooks, R. C. (2017). Do prevailing environmental factors influence human preferences for facial morphology? Behavioral Ecology, 28(5), 1217–1227. 10.1093/beheco/arx067

[ref34] Durante, K. M., & Li, N. P. (2009). Oestradiol level and opportunistic mating in women. Biology Letters, 5(2), 179–182. 10.1098/rsbl.2008.0709 19141415 PMC2665827

[ref35] Foo, Y. Z., Nakagawa, S., Rhodes, G., & Simmons, L. W. (2017). The effects of sex hormones on immune function: A meta-analysis. Biological Reviews, 92(1), 551–571. 10.1111/brv.12243 26800512

[ref36] Foo, Y. Z., Simmons, L. W., & Rhodes, G. (2017). Predictors of facial attractiveness and health in humans. Scientific Reports, 7(November 2016), 39731. 10.1038/srep39731 28155897 PMC5290736

[ref37] Friedland, R., Afary, J., Gardinali, P., & Naslund, C. (2016). Love in the Middle East: The contradictions of romance in the Facebook World. Critical Research on Religion, 4(3), 229–258. 10.1177/2050303216676523

[ref38] Gangestad, S. W., & Scheyd, G. J. (2005). The evolution of human physical attractiveness. Annual Review of Anthropology, 34, 523–548. https://doi.org/10.1146/

[ref39] Geniole, S. N., Denson, T. F., Dixson, B. J., Carré, J. M., & McCormick, C. M. (2015). Evidence from meta-analyses of the facial width-to-height ratio as an evolved cue of threat. PLoS ONE, 10(7), 1–18. 10.1371/journal.pone.0132726 PMC4504483 26181579

[ref40] Germine, L., Russell, R., Bronstad, P. M., Blokland, G. A. M., Smoller, J. W., Kwok, H., … Wilmer, J. B. (2015). Individual aesthetic preferences for faces are shaped mostly by environments, not genes. Current Biology, 25(20), 2684–2689. 10.1016/j.cub.2015.08.048 26441352 PMC4629915

[ref41] Gordon, M., & Lumley, T. (2020). forestplot: Advanced Forest Plot Using ‘grid’ Graphics. https://rdrr.io/cran/forestplot/

[ref42] Gu, Z., Eils, R., & Schlesner, M. (2016). Complex heatmaps reveal patterns and correlations in multidimensional genomic data. Bioinformatics, 32(18), 2847–2849. 10.1093/bioinformatics/btw313 27207943

[ref43] Gu, Z., Gu, L., Eils, R., Schlesner, M., & Brors, B. (2014). Circlize implements and enhances circular visualization in R. Bioinformatics, 30(19), 2811–2812. 10.1093/bioinformatics/btu393 24930139

[ref44] Hart, K. (2007). Love by arrangement: The ambiguity of ‘spousal choice’ in a Turkish village. Journal of the Royal Anthropological Institute, 13(2), 345–362. 10.1111/j.1467-9655.2007.00438.x

[ref45] Hausman, B. L. (1999). Ovaries to estrogen: Sex hormones and chemical femininity in the 20th century. Journal of Medical Humanities, 20(3), 165–176. 10.1023/A:1022926928578

[ref46] Henderson, A. J., Holzleitner, I. J., Talamas, S. N., & Perrett, D. I. (2016). Perception of health from facial cues. Philosophical Transactions of the Royal Society B: Biological Sciences, 371(1693). 10.1098/rstb.2015.0380 PMC4843618 27069057

[ref47] Holzleitner, I. J., Lee, A. J., Hahn, A. C., Kandrik, M., Bovet, J., Renoult, J. P., … Jones, B. C. (2019). Comparing theory-driven and data-driven attractiveness models using images of real women's faces. Journal of Experimental Psychology. Human Perception and Performance, 45(12), 1589–1595. 10.1037/xhp0000685 31556686

[ref48] Honarvar, B., Salehi, F., Barfi, R., Asadi, Z., Honarvar, H., Odoomi, N., … Lankarani, K. B. (2016). Attitudes toward and experience of singles with premarital sex: A population-based study in Shiraz, southern Iran. Archives of Sexual Behavior, 45(2), 395–402. 10.1007/s10508-015-0577-2 26334775

[ref49] Hönekopp, J. (2006). Once more: Is beauty in the eye of the beholder? Relative contributions of private and shared taste to judgments of facial attractiveness. Journal of Experimental Psychology: Human Perception and Performance, 32(2), 199–209. 10.1037/0096-1523.32.2.199 16634665

[ref50] Hunter, R. S. (1958). Photoelectric color difference meter. Journal of the Optical Society of America, 48(12), 985. 10.1364/JOSA.48.000985

[ref51] Johnston, V. S., Hagel, R., Franklin, M., Fink, B., & Grammer, K. (2001). Male facial attractiveness: Evidence for hormone-mediated adaptive design. Evolution and Human Behavior, 22, 251–267. 10.1145/3011286.3011307

[ref52] Jones, B. C., Hahn, A. C., & DeBruine, L. M. (2019). Ovulation, sex hormones, and women's mating psychology. Trends in Cognitive Sciences, 23(1), 51–62. 10.1016/j.tics.2018.10.008 30477896

[ref53] Jones, D., & Hill, K. (1993). Criteria of facial attractiveness in five populations. Human Nature, 4(3), 271–296. 10.1007/BF02692202 24214367

[ref54] Jones, A. L., & Jaeger, B. (2019). Biological bases of beauty revisited: The effect of symmetry, averageness, and sexual dimorphism on female facial attractiveness. Symmetry, 11(2), 279. 10.3390/sym11020279

[ref55] Kane, L., & Ashbaugh, A. R. (2017). Simple and parallel mediation: A tutorial exploring anxiety sensitivity, sensation seeking, and gender. The Quantitative Methods for Psychology, 13(3), 148–165. 10.20982/tqmp.13.3.p148

[ref56] Kleisner, K., Kočnar, T., Tureček, P., Stella, D., Akoko, R. M., Třebický, V., & Havlíček, J. (2017). African and European perception of African female attractiveness. Evolution and Human Behavior, 38(6), 744–755. 10.1016/j.evolhumbehav.2017.07.002

[ref57] Kleisner, K., Pokorný, Š., & Saribay, S. A. (2019). Toward a new approach to cross-cultural distinctiveness and typicality of human faces: The cross-group typicality/distinctiveness metric. Frontiers in Psychology, 10, 1–13. 10.3389/fpsyg.2019.00124 30766504 PMC6365443

[ref58] Koehler, N., Simmons, L. W., Rhodes, G., & Peters, M. (2004). The relationship between sexual dimorphism in human faces and fluctuating asymmetry. Proceedings of the Royal Society B: Biological Sciences, 271(suppl. 4), S233–S236. 10.1098/rsbl.2003.0146 PMC1810020 15252993

[ref59] Komori, M., Kawamura, S., & Ishihara, S. (2009). Averageness or symmetry: Which is more important for facial attractiveness? Acta Psychologica, 131(2), 136–142. 10.1016/j.actpsy.2009.03.008 19394585

[ref60] Komori, M., Kawamura, S., & Ishihara, S. (2011). Multiple mechanisms in the perception of face gender: Effect of sex-irrelevant features. Journal of Experimental Psychology: Human Perception and Performance, 37(3), 626–633. 10.1037/a0020369 20822297

[ref61] Kościński, K. (2007). Facial attractiveness: General patterns of facial preferences. Anthropological Review, 70(1), 45–79. 10.2478/v10044-008-0001-9

[ref62] Kościński, K. (2008). Facial attractiveness: Variation, adaptiveness and consequences of facial preferences. Anthropological Review, 71(1), 77–105. 10.2478/v10044-008-0012-6

[ref63] Kowner, R., & Ogawa, T. (1995). The role of rater's sex, personality, and appearance in judgments of facial beauty. Perceptual and Motor Skills, 81, 339–349. 10.2466/pms.1995.81.1.339

[ref64] Kramer, R. S. S. (2015). Facial width-to-height ratio in a large sample of commonwealth games athletes. Evolutionary Psychology, 13(1), 197–209. 10.1177/14747049150130011225714799

[ref65] Kramer, R. S. S., Mileva, M., & Ritchie, K. L. (2018). Inter-rater agreement in trait judgements from faces. PLoS ONE, 13(8), e0202655. 10.1371/journal.pone.0202655 30118520 PMC6097668

[ref66] Langlois, J., Kalakanis, L., Rubenstein, A., Larson, A., Hallam, M., & Smoot, M. (2000). Maxims or myths of beauty? A meta-analytical and theoretical review. Psychological Bulletin, 123(3), 390–423. 10.1037//0033-2909.126.3.390 10825783

[ref67] Law Smith, M. J., Perrett, D. I., Jones, B. C., Cornwell, R. E., Moore, F. R., Feinberg, D. R., … Hillier, S. G. (2006). Facial appearance is a cue to oestrogen levels in women. Proceedings. Biological Sciences/The Royal Society, 273(1583), 135–140. 10.1098/rspb.2005.3296 PMC1560017 16555779

[ref68] Lee, A. J., & Zietsch, B. P. (2011). Experimental evidence that women's mate preferences are directly influenced by cues of pathogen prevalence and resource scarcity. Biology Letters, 7(6), 892–895. 10.1098/rsbl.2011.0454 21697166 PMC3210674

[ref69] Lipson, S. F., & Ellison, P. T. (1996). Comparison of salivary steroid profiles in naturally occurring conception and non-conception cycles. Human Reproduction, 11(10), 2090–2096. 10.1093/oxfordjournals.humrep.a019055 8943508

[ref70] Little, A. C., Cohen, D. L., Jones, B. C., & Belsky, J. (2007). Human preferences for facial masculinity change with relationship type and environmental harshness. Behavioral Ecology and Sociobiology, 61(6), 967–973. 10.1007/s00265-006-0325-7

[ref71] Little, A. C, DeBruine, L. M., & Jones, B. C. (2013). Environment contingent preferences: Exposure to visual cues of direct male–male competition and wealth increase women's preferences for masculinity in male faces. Evolution and Human Behavior, 34(3), 193–200. 10.1016/j.evolhumbehav.2012.11.008

[ref72] Little, A. C., & Hancock, P. J. B. (2002). The role of masculinity and distinctiveness in judgments of human male facial attractiveness. British Journal of Psychology, 93(4), 451–464. 10.1348/000712602761381349 12519528

[ref73] Little, A. C., Jones, B. C., & DeBruine, L. M. (2011). Facial attractiveness: Evolutionary based research. Philosophical Transactions of the Royal Society B: Biological Sciences, 366(1571), 1638–1659. 10.1098/rstb.2010.0404 PMC3130383 21536551

[ref74] Little, A. C., Jones, B. C., Penton-Voak, I. S., Burt, D. M., & Perrett, D. I. (2002). Partnership status and the temporal context of relationships influence human female preferences for sexual dimorphism in male face shape. Proceedings of the Royal Society B: Biological Sciences, 269(1496), 1095–1100. 10.1098/rspb.2002.1984 PMC1691012 12061950

[ref75] Maestripieri, D., Klimczuk, A. C. E., Traficonte, D. M., & Wilson, M. C. (2014). A greater decline in female facial attractiveness during middle age reflects women's loss of reproductive value. Frontiers in Psychology, 5, 1–6. 10.3389/fpsyg.2014.00179 24592253 PMC3938111

[ref76] Marcinkowska, U. M., Kozlov, M. V., Cai, H., Contreras-Garduño, J., Dixson, B. J., Oana, G. A., … Rantala, M. J. (2014). Cross-cultural variation in men's preference for sexual dimorphism in women's faces. Biology Letters, 10(4), 4–7. 10.1098/rsbl.2013.0850 PMC4013689 24789138

[ref77] Marcinkowska, U. M., Rantala, M. J., Lee, A. J., Kozlov, M. V, Aavik, T., Cai, H., … Dixson, B. J. W. (2019). Women's preferences for men's facial masculinity are strongest under favorable ecological conditions. Scientific Reports, 9(3387), 1–10. 10.1038/s41598-019-39350-8 30833635 PMC6399235

[ref78] Marečková, K., Weinbrand, Z., Chakravarty, M. M., Lawrence, C., Aleong, R., Leonard, G., … Paus, T. (2011). Testosterone-mediated sex differences in the face shape during adolescence: Subjective impressions and objective features. Hormones and Behavior, 60(5), 681–690. 10.1016/j.yhbeh.2011.09.004 21983236

[ref79] Mazur, A., & Michalek, J. (1998). Marriage, divorce, and male testosterone. Social Forces, 77(1), 315–330. 10.2307/3006019

[ref80] McLellan, B., & McKelvie, S. J. (1993). Effects of age and gender on perceived facial attractiveness. Canadian Journal of Behavioural Science/Revue Canadienne Des Sciences Du Comportement, 25(1), 135–142. 10.1037/h0078790

[ref81] Mitteroecker, P., Windhager, S., Møller, G. B., & Schaefer, K. (2015). The morphometrics of ‘masculinity’ in human faces. PLoS ONE, 10(2), e0118374. 10.1371/journal.pone.0118374 25671667 PMC4324773

[ref82] Mogilski, J. K., & Welling, L. L. M. (2017). The relative importance of sexual dimorphism, fluctuating asymmetry, and color cues to health during evaluation of potential partners’ facial photographs: A conjoint analysis study. Human Nature, 28(1), 53–75. 10.1007/s12110-016-9277-4 27752965

[ref83] Mooradian, A. D., Morley, J. E., & Korenman, S. G. (1987). Biological actions of androgens. Endocrine Reviews, 8 (1)(1), 1–28. https://doi.org/10.1210 3549275 10.1210/edrv-8-1-1

[ref84] Moore, F. R., Cornwell, R. E., Law Smith, M. J., Al Dujaili, E. A. S., Sharp, M., & Perrett, D. I. (2011). Evidence for the stress-linked immunocompetence handicap hypothesis in human male faces. Proceedings of the Royal Society B: Biological Sciences, 278(1706), 774–780. 10.1098/rspb.2010.1678 PMC3030856 20843854

[ref85] Muñoz-Reyes, J. A., Iglesias-Julios, M., Pita, M., & Turiegano, E. (2015). Facial features: What women perceive as attractive and what men consider attractive. PLoS ONE, 10(7), 1–17. 10.1371/journal.pone.0132979 PMC4498779 26161954

[ref86] Nakamura, K., & Watanabe, K. (2019). Data-driven mathematical model of East-Asian facial attractiveness: The relative contributions of shape and reflectance to attractiveness judgements. Royal Society Open Science, 6(5). 10.1098/rsos.182189 PMC6549996 31218042

[ref87] Nowak, J., Pawłowski, B., Borkowska, B., Augustyniak, D., & Drulis-Kawa, Z. (2018). No evidence for the immunocompetence handicap hypothesis in male humans. Scientific Reports, 8(1), 1–11. 10.1038/s41598-018-25694-0 29743556 PMC5943526

[ref88] Özener, B. (2012). Facial width-to-height ratio in a Turkish population is not sexually dimorphic and is unrelated to aggressive behavior. Evolution and Human Behavior, 33(3), 169–173. 10.1016/j.evolhumbehav.2011.08.001

[ref89] Penna, V., Fricke, A., Iblher, N., Eisenhardt, S. U., & Stark, G. B. (2015). The attractive lip: A photomorphometric analysis. Journal of Plastic, Reconstructive and Aesthetic Surgery, 68(7), 920–929. 10.1016/j.bjps.2015.03.013 25921652

[ref90] Penton-Voak, I. S., & Chen, J. Y. (2004). High salivary testosterone is linked to masculine male facial appearance in humans. Evolution and Human Behavior, 25(4), 229–241. 10.1016/j.evolhumbehav.2004.04.003

[ref91] Penton-Voak, I. S., Jacobson, A., & Trivers, R. (2004). Populational differences in attractiveness judgements of male and female faces: Comparing British and Jamaican samples. Evolution and Human Behavior, 25(6), 355–370. 10.1016/j.evolhumbehav.2004.06.002

[ref92] Perrett, D. I., Lee, K. J., Penton-Voak, I. S., Rowland, D. A., Yoshikawa, S., Burt, D. M., … Akamatsu, S. (1998). Effects of sexual dimorphism on facial attractiveness. Nature, 394(6696), 884–887. 10.1038/29772 9732869

[ref93] Perrett, D. I., May, K. A., & Yoshikawa, S. (1994). Facial shape and judgements of female attractiveness. Nature, 368(6468), 239–242. 10.1038/368239a0 8145822

[ref94] Peters, M., Rhodes, G., & Simmons, L. W. (2008). Does attractiveness in men provide clues to semen quality? Journal of Evolutionary Biology, 21(2), 572–579. 10.1111/j.1420-9101.2007.01477.x 18179518

[ref95] Polo, P., Muñoz-Reyes, J. A., Pita, M., Shackelford, T. K., & Fink, B. (2019). Testosterone-dependent facial and body traits predict men's sociosexual attitudes and behaviors. American Journal of Human Biology, 31(3), 1–10. 10.1002/ajhb.23235 30884051

[ref96] Probst, F., Bobst, C., & Lobmaier, J. S. (2016). Testosterone-to-estradiol ratio is associated with female facial attractiveness. Quarterly Journal of Experimental Psychology, 69(1), 89–99. 10.1080/17470218.2015.1024696 25730636

[ref97] Rantala, M. J., Moore, F. R., Skrinda, I., Krama, T., Kivleniece, I., Kecko, S., & Krams, I. (2012). Evidence for the stress-linked immunocompetence handicap hypothesis in humans. Nature Communications, 3(394), 1–5. 10.1038/ncomms1696 PMC4355638 22353724

[ref98] R Core Team (2020). R: A language and environment for statistical computing. R Foundation forStatistical Computing, Vienna, Austria. https://www.R-project.org/.

[ref99] Rennels, J. L., Bronstad, P. M., & Langlois, J. H. (2008). Are attractive men's faces masculine or feminine? The importance of type of facial stimuli. Journal of Experimental Psychology. Human Perception and Performance, 34(4), 884–893. 10.1037/0096-1523.34.4.884 18665733

[ref100] Revelle, W. (2018). psych: Procedures for Personality and Psychological Research. Northwestern University. https://cran.r-project.org/package=psychVersion=1.8.12.

[ref101] Rhodes, G. (2006). The evolutionary psychology of facial beauty. Annual Review of Psychology, 57(1), 199–226. 10.1146/annurev.psych.57.102904.190208 16318594

[ref102] Rhodes, G., Chan, J., Zebrowitz, L. A., & Simmons, L. W. (2003). Does sexual dimorphism in human faces signal health? Proceedings. Biological Sciences/The Royal Society, 270(suppl.), S93–S95. 10.1098/rsbl.2003.0023 PMC1698019 12952647

[ref103] Rhodes, G., Hickford, C., & Jeffery, L. (2000). Sex-typicality and attractiveness: Are supermale and superfemale faces super-attractive? British Journal of Psychology, 91(1), 125–140. 10.1348/000712600161718 10717775

[ref104] Rhodes, G., Yoshikawa, S., Clark, A., Kieran, L., McKay, R., & Akamatsu, S. (2001). Attractiveness of facial averageness and symmetry in non-western cultures: In search of biologically based standards of beauty. Perception, 30(5), 611–625. 10.1068/p3123 11430245

[ref105] Rohlf, F. J. (2015). The tps series of software. Hystrix, 26(1), 1–4. 10.4404/hystrix-26.1-11264

[ref106] Rosseel, Y. (2012). lavaan: An R package for structural equation modeling. R package version 0.5-15. Journal of Statistical Software, 48(2), 1–36. https://econpapers.repec.org/article/jssjstsof/v_3a048_3ai02.htm%0Ahttp://www.jstatsoft.org/v48/i02/

[ref107] Ryder, H., Maltby, J., Rai, L., Jones, P., & Flowe, H. D. (2016). Women's fear of crime and preference for formidable mates: How specific are the underlying psychological mechanisms? Evolution and Human Behavior, 37(4), 293–302. 10.1016/j.evolhumbehav.2016.01.005

[ref108] Saribay, S. A., Biten, A. F., Meral, E. O., Aldan, P., Trebicky, V., & Kleisner, K. (2018). The Bogazici face database: Standardized photographs of Turkish faces with supporting materials. PLoS ONE, 13(2). 10.1371/journal.pone.0192018 PMC5812588 29444180

[ref109] Scheel, A. M., Tiokhin, L., Isager, P. M., & Lakens, D. (2020). Why hypothesis testers should spend less time testing hypotheses. Perspectives on Psychological Science. 10.1177/1745691620966795 PMC8273364 33326363

[ref110] Schneider, C. A., Rasband, W. S., & Eliceiri, K. W. (2012). NIH Image to ImageJ: 25 years of image analysis. Nature Methods, 9(7), 671–675. 10.1038/nmeth.2089 22930834 PMC5554542

[ref111] Schreiber, J. E., Singh, N. K., & Klatsky, S. A. (2005). Beauty lies in the ‘eyebrow’ of the beholder: A public survey of eyebrow aesthetics. Aesthetic Surgery Journal, 25(4), 348–352. 10.1016/j.asj.2005.05.002 19338830

[ref112] Scott, I. M., Clark, A. P., Josephson, S. C., Boyette, A. H., Cuthill, I. C., Fried, R. L., … Penton-Voak, I. S. (2014). Human preferences for sexually dimorphic faces may be evolutionarily novel. Proceedings of the National Academy of Sciences, 111(40), 14388–14393. 10.1073/pnas.1409643111 PMC4210032 25246593

[ref113] Scott, I. M. L., Pound, N., Stephen, I. D., Clark, A. P., & Penton-Voak, I. S. (2010). Does masculinity matter? The contribution of masculine face shape to male attractiveness in humans. PLoS ONE, 5(10), e13585. 10.1371/journal.pone.0013585 21048972 PMC2965103

[ref114] Shrout, P. E., & Fleiss, J. L. (1979). Intraclass correlations: uses in assessing rater reliability. Psychological Bulletin, 86(2), 420–428. http://www.ncbi.nlm.nih.gov/pubmed/18839484 18839484 10.1037//0033-2909.86.2.420

[ref115] Skrinda, I., Krama, T., Kecko, S., Moore, F. R., Kaasik, A., Meija, L., … Krams, I. (2014). Body height, immunity, facial and vocal attractiveness in young men. Naturwissenschaften, 101(12), 1017–1025. 10.1007/s00114-014-1241-8 25326093

[ref116] Smith, F. G., Jones, B. C., Debruine, L. M., & Little, A. C. (2009). Interactions between masculinity–femininity and apparent health in face preferences. Behavioral Ecology, 20(2), 441–445. 10.1093/beheco/arn141

[ref117] Sorokowski, P., Kościński, K., & Sorokowska, A. (2013). Is beauty in the eye of the beholder but ugliness culturally universal? Facial preferences of Polish and Yali (papua) people. Evolutionary Psychology, 11(4), 907–925. 10.1177/147470491301100414

[ref118] Sorokowski, P., Sorokowska, A., & Kras, D. (2013). Face color and sexual attractiveness: Preferences of Yali people of Papua. Cross-Cultural Research, 47(4), 415–427. 10.1177/1069397113485540

[ref119] Stephen, I. D., & McKeeganh, A. M. (2010). Lip colour affects perceived sex typicality and attractiveness of human faces. Perception, 39(8), 1104–1110. 10.1068/p6730 20942361

[ref120] Stephen, I. D., & Perrett, D. I. (2016). Color and face perception. In A. J. Elliot, M. D. Fairchild, & A. Franklin (Eds.), Handbook of Color Psychology (pp. 585–602). Cambridge University Press. 10.1017/cbo9781107337930.029

[ref121] Stephen, I. D., Scott, I. M. L., Coetzee, V., Pound, N., Perrett, D. I., & Penton-Voak, I. S. (2012). Cross-cultural effects of color, but not morphological masculinity, on perceived attractiveness of men's faces. Evolution and Human Behavior, 33(4), 260–267. 10.1016/j.evolhumbehav.2011.10.003

[ref122] Stirrat, M. R., & Perrett, D. I. (2010). Valid facial cues to cooperation and trust: Male facial width and trustworthiness. Psychological Science, 21(3), 349–354. 10.1177/0956797610362647 20424067

[ref123] Strom, M. A., Zebrowitz, L. A., Zhang, S., Bronstad, P. M., & Lee, H. K. (2012). Skin and bones: The contribution of skin tone and facial structure to racial prototypicality ratings. PLoS ONE, 7(7), e41193. 10.1371/journal.pone.0041193 22815966 PMC3399873

[ref124] Strzałko, J., & Kaszycka, K. A. (1992). Physical attractiveness: Interpersonal and intrapersonal variability of assessments. Biodemography and Social Biology, 39(1–2), 170–176. 10.1080/19485565.1992.9988813 1514121

[ref125] Swaddle, J. P., & Reierson, G. W. (2002). Testosterone increases perceived dominance but not attractiveness in human males. Proceedings of the Royal Society B: Biological Sciences, 269(1507), 2285–2289. 10.1098/rspb.2002.2165 PMC1691166 12495494

[ref126] Thornhill, R., & Gangestad, S. W. (1999). Facial attractiveness. Trends in Congnitive Sciences, 3(12), 452–460. 10.1016/s1364-6613(99)01403-5 10562724

[ref127] Thornhill, R., & Gangestad, S. W. (2006). Facial sexual dimorphism, developmental stability, and susceptibility to disease in men and women. Evolution and Human Behavior, 27(2), 131–144. 10.1016/j.evolhumbehav.2005.06.001

[ref128] Thornhill, R., & Grammer, K. (1999). The body and face of woman: One ornament that signals quality? Evolution and Human Behavior, 20(2), 105–120. 10.1016/S1090-5138(98)00044-0

[ref129] Třebický, V., Fialová, J., Kleisner, K., Roberts, S. C., Little, A. C., & Havlíček, J. (2015). Further evidence for links between facial width-to-height ratio and fighting success: Commentary on Zilioli et al. (2014). Aggressive Behavior, 41(4), 331–334. 10.1002/ab.21559 25236530

[ref130] Třebický, V., Fialová, J., Stella, D., Štěrbová, Z., Kleisner, K., & Havlíček, J. (2018). 360 degrees of facial perception: Congruence in perception of frontal portrait, profile, and rotation photographs. Frontiers in Psychology, 9(2405), 1–11. 10.3389/fpsyg.2018.02405 30581400 PMC6293201

[ref131] Třebický, V., Stirrat, M., & Havlíček, J. (2019). Fighting assessment. Encyclopedia of Evolutionary Psychological Science, 1–11. 10.1007/978-3-319-16999-6_2738-1

[ref132] van Bokhoven, I., van Goozen, S. H. M., van Engeland, H., Schaal, B., Arseneault, L., Séguin, J. R., … Tremblay, R. E. (2006). Salivary testosterone and aggression, delinquency, and social dominance in a population-based longitudinal study of adolescent males. Hormones and Behavior, 50(1), 118–125. 10.1016/j.yhbeh.2006.02.002 16631757

[ref133] van den Berghe, P. L., & Frost, P. (1986). Skin color preference, sexual dimorphism and sexual selection: A case of gene culture co-evolution? Ethnic and Racial Studies, 9(1), 87–113. 10.1080/01419870.1986.9993516

[ref134] Vitzthum, V. J. (2009). The ecology and evolutionary endocrinology of reproduction in the human female. American Journal of Physical Anthropology, 140(S49), 95–136. 10.1002/ajpa.v140.49s 19890865

[ref135] Vitzthum, V. J., Bentley, G. R., Spielvogel, H., Caceres, E., Thornburg, J., Jones, L., … Chatterton, R. T. (2002). Salivary progesterone levels and rate of ovulation are significantly lower in poorer than in better-off urban-dwelling Bolivian women. Human Reproduction, 17(7), 1906–1913. 10.1093/humrep/17.7.1906 12093859

[ref136] Wagatsuma, H. (1967). The social perception of skin color in Japan. Daedalus, 96(2), 407–443. https://www.jstor.org/stable/20027045

[ref137] Whitehouse, A. J. O., Gilani, S. Z., Shafait, F., Mian, A., Tan, D. W., Maybery, M. T., … Eastwood, P. (2015). Prenatal testosterone exposure is related to sexually dimorphic facial morphology in adulthood. Proceedings of the Royal Society B: Biological Sciences, 282(1816). 10.1098/rspb.2015.1351 PMC4614768 26400740

[ref138] Worthman, C. M. (1995). Hormones sex gender. Annual Review of Anthropology, 24, 593–617.

[ref139] Zebrowitz, L. A., Wang, R., Bronstad, P. M., Eisenberg, D. T. A., Undurraga, E. A., Reyes-García, V., & Godoy, R. A. (2012). First impressions from faces among U.S. and culturally isolated Tsimane’ people in the Bolivian rainforest. Journal of Cross-Cultural Psychology, 43(1), 119–134. 10.1177/0022022111411386

